# Intermittent Theta Burst Stimulation Over Ventral Premotor Cortex or Inferior Parietal Lobule Does Not Enhance the Rubber Hand Illusion

**DOI:** 10.3389/fnins.2018.00870

**Published:** 2018-11-23

**Authors:** Alessandro Mioli, Marco D’Alonzo, Giovanni Pellegrino, Domenico Formica, Giovanni Di Pino

**Affiliations:** ^1^Research Unit of Neurophysiology and Neuroengineering of Human-Technology Interaction, Università Campus Bio-Medico, Rome, Italy; ^2^IRCCS Fondazione Ospedale San Camillo, Venice, Italy

**Keywords:** neuromodulation, transcranial magnetic stimulation, embodiment, body ownership, body representation

## Abstract

An enhanced sense of prosthesis ownership may be the key for higher amputees’ quality of life. In this study in 28 healthy subjects, neuronavigated intermittent Theta Burst Stimulation (iTBS) delivered over the right ventral premotor cortex or inferior parietal lobule has been tested, compared to sham stimulation, to enhance embodiment in the rubber hand illusion paradigm. Neuromodulation of both areas did not result in an enhancement of embodiment, as assessed by the results collected from a self-evaluation questionnaire for the extent of self-attribution of the rubber hand and proprioceptive drift. In all cases, the difference between synchronous and asynchronous stroking confirms the successful induction of the illusion. It may be speculated that the low consistency of iTBS over brain regions other than primary motor cortex may account for the absence of effect, suggesting to test other neuromodulating techniques, acting on cortical networks different from the ones sensitive to iTBS to enhance artificial hand embodiment.

## Introduction

Among the cognitive functions the establishment of the representation of the body is one of the most investigated while, in bionics, the cognitive aspects of prosthetics are attracting great attention and funds. Hand amputation distorts body representation so that an enhanced sense of prosthesis ownership may be the key for a successful treatment. Embodiment, the process of feeling not-owned limbs as part of our body, rises from the activation of premotor and multisensory associative areas within the frontoparietal network (Ehrsson et al., 2004), thus inducing plastic changes in this network should impact on embodiment. Excitatory protocols of repetitive transcranial magnetic stimulation (rTMS) are effective in enhancing plasticity and have behavioral effects in healthy and impaired subjects (Di Pino et al., 2014b), but they have never been used to boost prosthesis embodiment so far (Di Pino et al., 2014a).

A reliable way to induce and test embodiment of an artificial hand is by mean of the rubber hand illusion (RHI) paradigm (Botvinick and Cohen, 1998). A rubber hand is located directly in front of the participant, parallel to their real hand. The latter is hidden from the participant’s sight. The illusion is induced when the hidden real hand is brush-stroked synchronously (in time) and congruently (in space) with the visible rubber hand.

A bayesian bottom-up integration process of convergent multisensory inputs enables a sense of ownership for the rubber hand, by integrating it into the participant’s body schema (Armel and Ramachandran, 2003). Moreover, a distortion of proprioception also emerges, because the participant tends to estimate the position of their real hand closer to the rubber hand than it actually is (proprioceptive drift) (Botvinick and Cohen, 1998).

Electroencephalography (EEG) (Zeller et al., 2015; Rao and Kayser, 2017) and functional magnetic resonance imaging (fMRI) studies (Ehrsson et al., 2004; Brozzoli et al., 2012; Gentile et al., 2013) link the neural correlates of the RHI induction to a frontoparietal network where premotor and intraparietal sulcus areas tightly interact. These areas contain multimodal neurons that are able to integrate visual and somatosensory information, probably underpinning the representation of our corporeal space (Braun et al., 2018).

In this study in healthy subjects, intermittent theta burst stimulation (iTBS), a facilitatory rTMS protocol, has been used to enhance the excitability of the right ventral premotor cortex (rPMv) or inferior parietal lobule (rIPL). The leading hypothesis was that a facilitatory neuromodulation of those areas would have enhanced the embodiment of the rubber hand. We formulated this hypothesis because we thought that the administration of iTBS over those areas would have changed their excitability and interplay, resulting in an enhancement of body ownership over the artificial limb. In this case, our approach would have been useful in the future to enhance prosthesis embodiment in amputees. TBS has been chosen because of its average strong efficacy and its favorable ratio between time need to neuromodulate (2–3 min) and length of the effect (20–30 min) (Huang et al., 2005; Suppa et al., 2016).

Previously, amputees have been reported able to experience the RHI with an enhancement of embodiment over the fake hand (assessed through a self-evaluation questionnaire of the body-ownership index) and drift score of about 50% compared to the control condition (Ehrsson et al., 2008). Thus, in order to achieve a valuable change of embodiment, we targeted half of the reported mean shift, i.e., a 25% increase of the body-ownership index assessed by the self-evaluation questionnaire, which was chosen as the main expected outcome of the study.

## Materials and Methods

Twenty-eight participants (sex:16F, 12M; age: 26.68, *SD*: 4.66, range: 21–39. Four of the participants were left-handed as assessed with the Edinburgh Handedness Inventory), after signing a written informed consent, took part in the study. The number of enrolled subjects was determined considering that: (i) based on the RHI Index data distribution from (Abdulkarim and Ehrsson, 2016) (Mean: 2.1, SD: 1.15), to show a 25% mean shift, achieving an effect size of about 0.5 and a power of about 0.5, 25 subjects were needed, (ii) two subjects were excluded from the study because they did not complete all the experimental sessions and another one for being unable to follow experimenter’s instructions.

**FIGURE 1:**
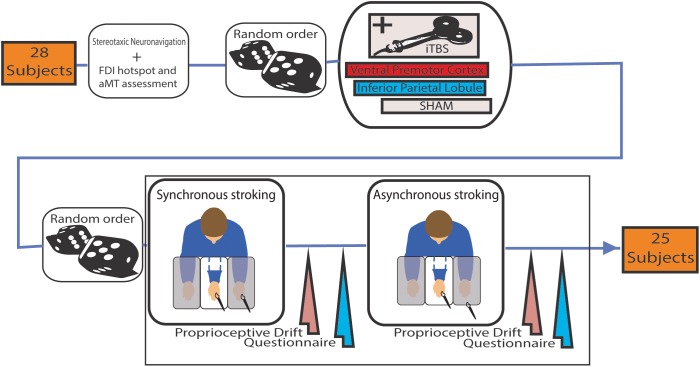
Schema of the experimental procedure of the study. Intermittent theta burst stimulation (iTBS) was administered before both Synchronous or Asynchronous stroking (in a random order) using paintbrushes. In the latter, the temporal mismatch of the stimuli was about 0.5 s.

Participants underwent three sessions of neuromodulation (iTBS over rPMv; iTBS over IPL and SHAM) in a random order. Each session consisted of neuronavigation, neuromodulation (i.e., iTBS), synchronous and asynchronous (control condition) RHI (Figure 1). In each session synchronous and asynchronous RHI order was randomized and both delivered in a range of 10–20 min when the effect of iTBS was reported to be at his peak (Huang et al., 2005).

Participants were placed in front of a purposely assembled structure with three separated compartments below a two-way mirror. Each compartment had its own illumination system, so that the experimenter could choose whether or not the participant could see its content.

The participant’s forearms were placed inside the two lateral compartments while shoulders were covered by a black cloak. The rubber hand was placed in the central compartment 15 cm medially to the participant’s left hand, and it was made visible by turning on the central compartment light. Once the rubber hand was visible, the experimenter started to synchronously or asynchronously (depending on the current condition) strike the participant’s hand and the rubber hand with paintbrushes for 90 s.

A 9-item questionnaire and the proprioceptive drift (Botvinick and Cohen, 1998) were collected to measure the effectiveness of RHI induction. The questionnaire required the participants to rate the strength of their agreement or disagreement with nine statements by using a 7-point Likert scale. Three of the statements (i.e., illusion statements) referred to the extent of self-attribution of the rubber hand during the trial. The other six statements (i.e., control statements) served as controls for compliance, suggestibility, and “placebo effect” (Supplementary Figure S1). Questionnaire outcome was evaluated through the RHI Index (mean of the three illusion questions minus mean of the six control questions) (Abdulkarim and Ehrsson, 2016). For the proprioceptive drift assessment, participants had to report a number on a measuring tape reflected on the two-way mirror that corresponded to the perceived location of their left index finger. The proprioceptive drift was calculated by subtracting the score obtained before each RHI procedure from the score collected right after it, where positive values indicate a drift toward the rubber hand in participants’ sense of the hand position.

Magnetic stimulation was performed with a biphasic magnetic stimulator (Duomag XT-100, Deymed, Hronov, Czechia) and a 9 cm figure-of-eight coil. Active motor threshold (aMT) was determined as the minimum single-pulse intensity required to produce at least five out of 10 MEPs greater than 200 μV in the left first dorsal interosseous (FDI), while the subject was maintaining a voluntary contraction of about 20% of maximum force. iTBS pattern was produced with three pulses of stimulation given at 50 Hz, repeated every 200 ms for 2 s, then a pause of 8 s for a total of 600 pulses and 190 s (Huang et al., 2005). The stimulus intensity was set at 80% of aMT.

Real stimulation was delivered either over PMv or over IPL on the right hemisphere and the RHI tested on the contralateral left hand, since right hemisphere is prevalent in the RHI task and in the establishment of body ownership (Meador et al., 2000; Ehrsson et al., 2005; Ocklenburg et al., 2011). Stimulation points on the scalp were found at the beginning of the first session referring to Talairach coordinates, corresponding to rPMv (*x* = 52, *y* = 10, *z* = 24) and rIPL (*x* = 56, *y* = -27, *z* = 37), with the help of an optoelectronic neuronavigation system (SofTaxic 2.0, EMS, Bologna, Italy). Sham iTBS was done over the vertex with the coil placed perpendicular to the scalp. Sessions differed at least 48 h from each other.

The three-session, single-blind, sham-controlled, counterbalanced cross-over experimental protocol was conducted in accordance to the ethical standards of the Declaration of Helsinki and was approved by the relevant Ethics Committee.

3 × 2 repeated measures ANOVA was employed separately for RHI Index and proprioceptive drift, with the factors *Neuromodulation* (Sham vs. rPMv vs. rIPL) and *Synchrony* (Synchronous vs. Asynchronous).

## Results

**FIGURE 2:**
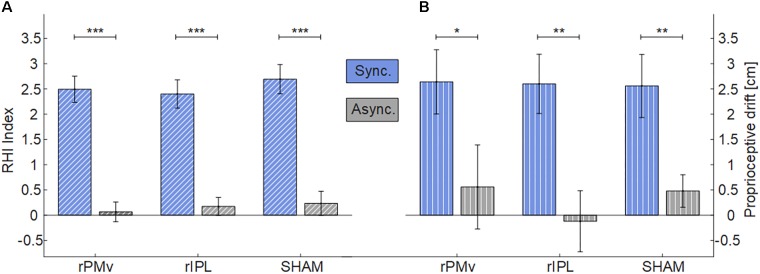
Mean and standard error of RHI Index **(A)** and Proprioceptive drift **(B)** in each neuromodulating condition. (^∗^*p* < 0.05; ^∗∗^*p* < 0.01; ^∗∗∗^*p* < 0.001). rPMv = right ventral premotor cortex; rIPL = right inferior parietal lobule.

Data were normally distributed (Shapiro-Wilks, *p* > 0.05) (Supplementary Figure S2). For both RHI Index and proprioceptive drift there was the main effect of *Synchrony* (Figure 2) [RHI Index: *F*(1,24) = 62.738, *p* < 0.001); proprioceptive drift: *F*(1,24) = 24.554, *p* < 0.001)], while there was not effect of *Neuromodulation* [RHI Index: *F*(2,48) = 0.527, *p* = 0.594); proprioceptive drift: *F*(2,48) = 0.243, *p* = 0.785)] or interaction *Synchrony* × *Neuromodulation* [RHI Index: *F*(2,48) = 0.323, *p* = 0.726); proprioceptive drift: *F*(2,48) = 0.227, *p* = 0.798)]. Planned comparisons with Holm corrected *t*-tests between synchronous and asynchronous condition were significant in all neuromodulating conditions (RHI Index: all *p* < 0.001; proprioceptive drift: all *p* < 0.021).

## Discussion

This is the first study where a facilitatory rTMS protocol has been employed to foster artificial hand embodiment. Hitherto, rTMS has been exploited only with plasticity-decreasing inhibitory protocols to induce virtual lesions. Indeed, 1 Hz rTMS over IPL has been reported to reduce proprioceptive drift (Kammers et al., 2009), while over the primary motor cortex (M1) to reduce the real hand ownership, making participants more prone to embody the rubber hand (Fossataro et al., 2018). The real hand disembodiment was due to the down-regulation of its motor pathway after the “virtual lesion” of M1. This finding was not suited to be exploited for our aim because we target to increase a behavior (embodiment), thus we have chosen a facilitatory protocol, and because our final aim is the enhancement of prosthesis embodiment in amputees who have not their real own hand to disembody.

Despite a large sample size and a well-controlled within-subject design, iTBS over rPMv or rIPL did not result in an enhancement of embodiment. No significant effects of the stimulation were found on two different investigated measures: the readout of the questionnaire, which is more informative of changes in ownership, the proprioceptive drift which is more specific for a spatial update of the sense of hand’s position (Rohde et al., 2011). The presence of the well-known difference of induced embodiment between synchrony and asynchrony in stroking confirms that the illusion was successfully induced in all cases.

The interpretation of these results can be twofold. Embodiment of a fake limb cannot be increased with non-invasive brain stimulation techniques due to a possible ceiling effect. However, the recent reports of a slight increase of proprioceptive drift and subjective experience of body ownership with transcranial direct current stimulation (Convento et al., 2018; Lira et al., 2018) may suggest that iTBS could be not the better suited neuromodulating protocol for this purpose. iTBS was chosen because it is short and effective, but differences in the individual network sensitivity to the magnetic pulse (Hamada et al., 2012) and in individual functional connectivity (Nettekoven et al., 2015) make iTBS efficacy highly variable across subjects and, especially, across brain regions other than primary motor cortex (Suppa et al., 2016).

Supported by the methodological strengths of the study, we can conclude for an absence of effect of iTBS, over the main areas responsible of embodiment induction. We tested only typical iTBS parameters and right hemisphere. However, what we see as more promising to enhance hand prosthesis embodiment and prosthesis users’ quality of life is to test neuromodulation techniques acting on cortical circuitry other than the one sensitive to iTBS (e.g., Paired Associative Stimulation).

## Ethics Statement

The protocol of the study was approved by the Ethics Committee of Università Campus Bio-Medico di Roma (EMBODY protocol). All subjects gave written informed consent. This study was carried out in accordance with the Declaration of Helsinki and future amendments.

## Author Contributions

AM prepared and revised the manuscript, collected and analyzed the data, and interpreted the results. MD collected and analyzed the data. GP revised the manuscript and analyzed the data. DF analyzed the data. GD revised the manuscript, developed the study concept and designed, and interpreted the results.

## Conflict of Interest Statement

The authors declare that the research was conducted in the absence of any commercial or financial relationships that could be construed as a potential conflict of interest.
