# Supplementary material for: Intermittent Theta Burst Stimulation Over Ventral Premotor Cortex or Inferior Parietal Lobule Does Not Enhance the Rubber Hand Illusion
Source: Front Neurosci. 2018 Nov 23;12:870. doi: 10.3389/fnins.2018.00870 (PMC6265367; doi:10.3389/fnins.2018.00870)
Supplement: Supplementary file 1 [file Data_Sheet_1.docx]

Supplementary Material

Intermittent theta burst stimulation over ventral premotor cortex or inferior parietal lobule does not enhance the rubber hand illusion

Alessandro Mioli^1^, Marco D’Alonzo^1^, Giovanni Pellegrino^2^, Domenico Formica^1^, Giovanni Di Pino^1*^

^1^Research Unit of Neurophysiology and Neuroengineering of Human-Technology Interaction, University Campus Bio-Medico di Roma, Rome, Italy

^2^ IRCCS Fondazione Ospedale San Camillo, Venice, Italy

*** Correspondence:**Giovanni Di Pino
[g.dipino@unicampus.it](mailto:g.dipino@unicampus.it)

## Supplementary Figures
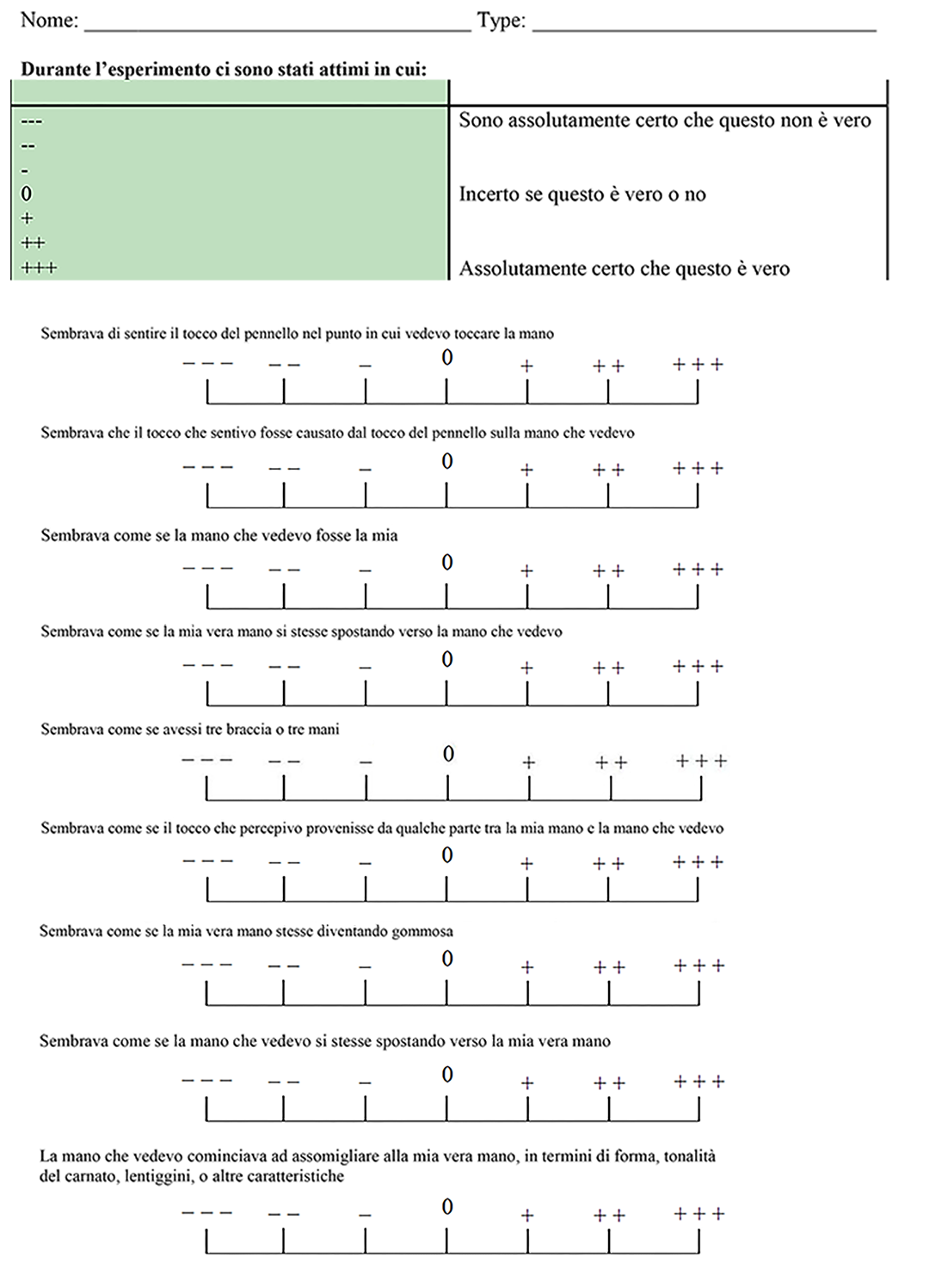


**Supplementary Figure 1:** Italian version (translated) of the questionnaire originally developed by Botvinick and Cohen (1998). The first three statements refer to the extent of self-attribution of the rubber hand during the trial. The other six, are control statements.


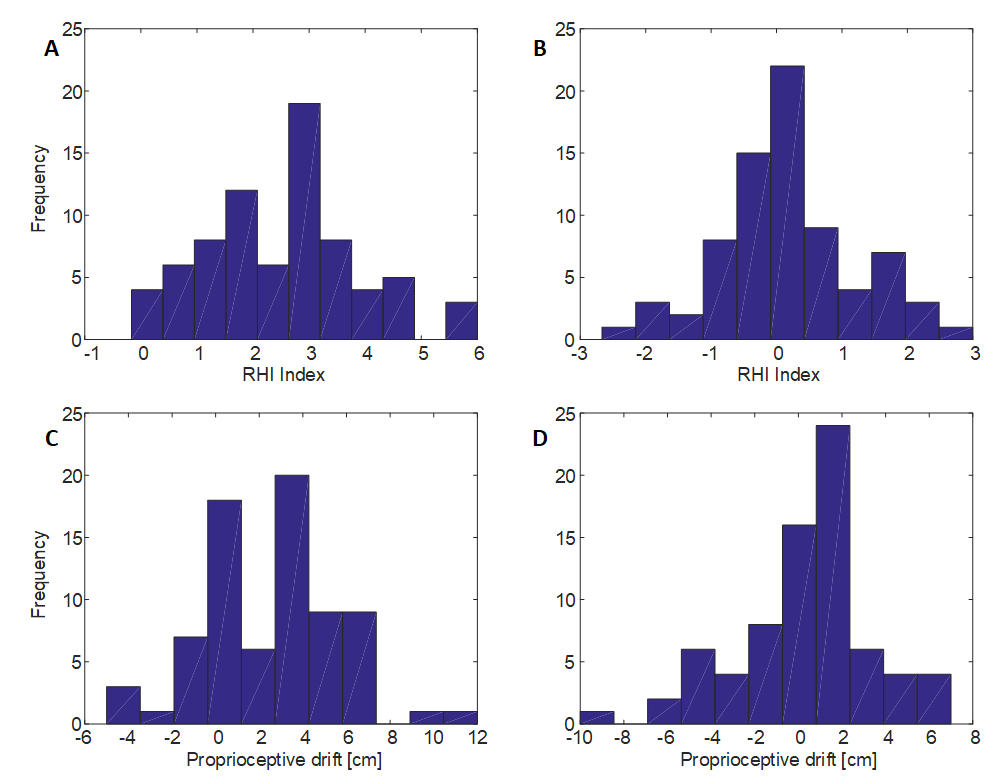


**Supplementary Figure 2:** Distribution of data of RHI Index in synchronous (A) and asynchronous (B) condition and of Proprioceptive drift in synchronous (C) and asynchronous (D) condition.
